# Poly(*meta/para*-Terphenylene-Methyl Piperidinium)-Based Anion Exchange Membranes: The Effect of Backbone Structure in AEMFC Application

**DOI:** 10.3390/membranes10110329

**Published:** 2020-11-05

**Authors:** T. S. Mayadevi, Seounghwa Sung, Listo Varghese, Tae-Hyun Kim

**Affiliations:** 1 Organic Material Synthesis Laboratory, Department of Chemistry, Incheon National University, 119 Academy-ro, Yeonsu-gu, Incheon 22012, Korea; mayadevits24@inu.ac.kr (T.S.M.); tmdghk712@naver.com (S.S.); listovarghese@gmail.com (L.V.); 2 Research Institute of Basic Sciences, Incheon National University, 119 Academy-ro, Yeonsu-gu, Incheon 22012, Korea

**Keywords:** *para/meta*-terphenyl, Friedel–Crafts polycondensation, free volume, anion exchange membrane

## Abstract

A series of poly(*meta/para*-terphenylene-methyl piperidinium)-based anion exchange membranes devoid of benzylic sites or aryl ether bonds, that are vulnerable to degradation by hydroxide ions, are synthesized and investigated for their application as novel anion exchange membranes. The copolymers are composed of both linear *para*-terphenyl units and kink-structured *meta*-terphenyl units. The *meta*-connectivity in terphenyl units permits the polymer backbones to fold back, maximizing the interactions among the hydrocarbon polymer chains and enhancing the peripheral formation of ion aggregates, due to the free volume generated by the kink structure. The effects of the copolymer composition between *para*-terphenyl and *meta*-terphenyl on the morphology and the electrochemical and physicochemical properties of the corresponding polymer membranes are investigated.

## 1. Introduction

As environmentally friendly power sources, anion exchange membrane fuel cells (AEMFCs) offer various advantages, including the ability to use a wide range of non-noble metal catalysts and faster oxidation reduction rates than the proton exchange membrane fuel cells (PEMFCs) currently in use [1,2,3,4,5,6,7]. The commercial application of AEMFC, however, has been impeded by the lack of materials for anion exchange membranes (AEMs) that have both sufficient ion (OH^−^) conductivity and adequate mechanical or chemical stabilities.

Through significant efforts, including controlling morphology of polymers [8,9] or changing the polymer structure from comb-type to spacer-type [10,11,12], as well as tuning the cationic head groups [13,14,15], a reasonably high conductivity has been achieved for the development of AEMs. Nevertheless, alkaline stability improvement has remained a challenge in the development of AEMs for commercialization of AEMFC [16,17].

It is recognized that both the conducting head groups and the polymer backbones are responsible for the stability of AEMs, and the general degradation pathways are well understood [18]. In the case of polymer main backbones, most commonly used polymers have the aryl ether (C–O) linkages in the backbone structure, and hence are now considered vulnerable to degradation by the nucleophilic attack of hydroxide ions on the *ipso* carbon position in oxyphenylene groups. Other undesired side reactions such as the Hofmann-*β*-elimination and ylide formation are also possible in conventional polymer backbones such as poly(aryl ether sulfone), poly(phenylene oxide)s and poly(aryl ether ketone), where the unstable benzylic positions are combined with the above mentioned aryl ether linkages [19,20,21,22,23,24,25].

To improve alkaline stability, it was therefore necessary to design the polymer backbone without aryl ether linkages.

**Figure 1:**
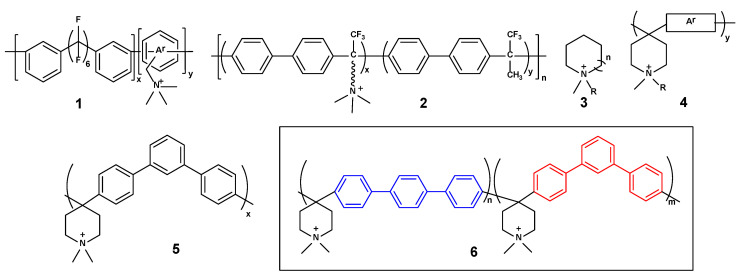
Structure of polyarylene backbone-type anion exchange membranes (AEMs) as poly(arylene perfluoroalkylene)s (**1**) [26] and poly(biphenyl alkylene)s (**2**) [27], hetero-cycloaliphatic-type conducting head groups (**3**) [29], and poly(arylene piperidinium)s (**4**) [16], poly(*meta*-terphenylene-methyl piperidinium) (**5**) [35] reported in the literature, and poly(*meta*/*para*-terphenylene-methyl piperidinium) (**6**) developed in this study.

Miyatake et al. introduced a new family of quaternized poly(arylene perfluoroalkylene)s, (QPAFs) **1** (Figure 1), having perfluoroalkylene groups and aromatic rings, avoiding heteroatom linkages in the polymer backbone [26]. Even though these kinds of molecular varieties have shown better stabilities in AEMs, the quaternary ammonium (QA) cations placed in the typical benzylic positions have remained a problem, due to loss of the cation as a conducting head group.

Meanwhile, Bae et al. developed a new synthetic strategy for AEM polymers, using the acid-catalyzed Friedel–Crafts polycondensation reaction between biphenyl and trifluoromethyl ketones to produce poly(biphenyl alkylene)s with the QA groups on the long alkyl side chain **2** (Figure 1), instead of the benzylic position, which demonstrated an enhanced stability along with a high conductivity for the corresponding membranes [27,28].

Marino and Kreuer have recently investigated the stability and degradation trends of various cationic head groups and demonstrated the exceptional stability of hetero-cycloaliphatic compounds **3** (Figure 1) [29,30]. This opened a new area for developing a wide variety of alkaline stable polymers with hetero-cycloaliphatic backbones with cationic head groups on the polymer backbone structures. In particular, *N,N*-dimethylpiperidinium is now considered one of the most stable (and highly conductive) conducting head groups, due to a lower ring strain and conformational constraints (ring structures which impose conformational constraints are known to increase the energy of the transition state for both substitution and elimination reactions [29,30]).

To combine the two latter strategies (acid-catalyzed Friedel–Crafts polycondensation reaction between biphenyl and trialkyl ketones and the formation of hetero-cyclic backbone structure having *N,N*-dimethylpiperidinium conducting head group), Jannasch et al. developed a new class of polymers that are poly(arylene piperidinium)s **4** (Figure 1) as highly stable and conductive anion exchange membranes, following the acid-catalyzed Friedel–Crafts polycondensation reaction [16,31]. The same group has also investigated the effect of backbone geometry on the properties of poly(terphenylene)-based AEMs and found that the more flexible *meta*-terphenyl backbone contributed greater conductivity than the rigid *para*-terphenyl units [32]. A *meta*-terphenyl as a monomer has benefits over linear and rigid *para*-terphenyl because the former with its kink structure exhibits the distorted spatial configuration able to regulate the morphology of the corresponding polymer membranes [25].

In fact, the geometry of the polymer structure can play a key role in controlling the microstructure of the polymers, which helps to facilitate hydroxide ion transport and hence improve fuel cell performance. Notably, an increase in the flexibility of the polymer backbone has a positive effect on the polymer’s mobility and helps to form well-developed ionic channels, hence enhancing ion conductivity. The higher flexibility of the polymer backbone can also facilitate water uptake of the membranes, which may affect the stability of AEMs by diluting the concentration of reactive hydroxide ions toward the nucleophilic attack [32,33,34].

Recently, QA-functionalized poly(*meta*-terphenylene-methyl piperidinium)-based anion exchange membranes (QMter-co-Mpi) **5** (Figure 1) having the non-aryl ether-type backbone structure were also developed by He et al. for high-performance water electrolysis application [35]. The introduction of twisted *meta*-terphenyl monomer in the polymer backbone efficiently built the ion transport channel and resulted in a comparably high conductivity but has not been investigated for AEMFC application.

Another example of using poly(*meta*-terphenylene-alkyl piperidinium)s as an AEM material introduces a long hydrophobic alkyl group grafted onto the side chain of the piperidinium group in order to inhibit problems such as swelling of the corresponding membranes caused by high water uptake in this type of kink-structured polymer [25]. Despite the reduced swelling, the long alkyl side chain on the conducting head group (alkyl piperidinium in this case) has an additional potential drawback of reducing the alkaline stability of the corresponding polymer membranes [36].

We report herein for the first time the development of the copolymers between *para*-terphenyl and *meta*-terphenyl **6** (Figure 1) units with different ratios to balance the conformational changes, due to the twisted/kink and rigid structures in the corresponding copolymer membranes, together with the cyclic methyl piperidium in the backbone as a conducting head group, and we investigate the possibility of applying these poly(*meta*/*para*-terphenylene-piperidinium)-based AEMs to AEMFC application. The effects of the copolymer composition between *para*-terphenyl and *meta*-terphenyl on the morphology and electrochemical and physicochemical properties of the corresponding polymer membranes **6** are investigated in this study.

## 2. Materials and Methods

### 2.1. Materials

*meta*-Terphenyl (*m*-terphenyl) (99%, Alfa Aesar, Seoul, Korea), *para*-terphenyl (*p*-terphenyl) (>99%, TCI, Seoul, Korea), *N*-methyl-4-piperidone (>98%, TCI, Seoul, Korea), iodomethane (99%, Sigma Aldrich, Yongin, Korea), trifluoromethane sulfonicacid (TFSA) (99%, Acros Organics, Seoul, Korea), and trifluoroacetic acid (>99%, TCI, Seoul, Korea) were purchased and used as they were unless mentioned. The *p*- and *m*-terphenyls were recrystallized from ethyl acetate and isopropyl alcohol respectively, prior to the polymerization. *N*-Methyl-4-piperidone was used after purification by vacuum distillation. HPLC grade dichloromethane (DCM) and dimethyl sulfoxide (DMSO) were used as received throughout the synthetic procedures. K_2_CO_3_, NaOH, KOH, and other solvents such as methanol, isopropyl alcohol, and ether were purchased from Daejung Metals & Chemicals Co., Ltd. (Shiheung, Korea) and used as received.

#### Synthesis of Poly(*meta/para*-terphenylene-methyl piperidinium) Copolymers with Various Contents of *m*-terphenyl Units, *m-p*-MP-y, **6**

The synthesis of poly(*meta/para*-terphenylene-methyl piperidinium)s, *m-p*-MP-y, where y represents the percentage ratio of *m*-terphenyl content, was carried out by reacting *m/p*-terphenyl with *N*-methyl-4-piperidone through the acid-catalyzed Friedel–Crafts polymerization reactions [16,37,38], followed methylation of the piperidine. Three sets of copolymers with different *m*-terphenyl molar ratios of 20%, 50%, and 60% were reacted with *N*-methyl-4-piperidone to give copolymers with three different *meta-*terphenylene-piperidinium units, and the copolymers were termed *m*-*p*-MP-20, *m*-*p*-MP-50 and *m*-*p*-MP-60, respectively.

The following is the general procedure for the synthesis of *m*-*p*-MP-20 as an example: the *N*-methyl-4-piperidone (1.00 g, 8.84 mmol), *m*-terphenyl (0.41 g, 1.77 mmol) and *p*-terphenyl (1.63 g, 7.07 mmol) were taken in a 25 mL round bottom flask equipped with a magnetic stirrer and dissolved in 6 mL of DCM. The TFSA (7.82 mL, 88.37 mmol) was then added to this solution dropwise under an ice bath. The reaction vessel was kept in an ice bath during polymerization to inhibit the elimination products. The color of the reaction mixture changed gradually from light yellow to dark brown. The reaction was stopped when the viscosity of the reaction mixture had increased to a limit which prevented normal magnetic stirring. The mixture was then poured into a 5 M aqueous NaOH solution yielding a white fibrous solid. The precipitate was then chopped and stirred in the same alkaline solution and washed several times with deionized water. The product was then filtered and dried overnight in an oven at 80 °C to yield poly(*meta*/*para*-terphenylene-piperidine), *m*-*p*-Pip-20 as a white solid (2.75 g, 91%); *δ*_H_ (400 MHz, DMS-*d*_6_/TFA) 7.91–7.36 (12H, m, H_4,5,6,7,8,9_), 3.52 (2H, broad signal, H_2_), 3.20 (2H, broad signal, H_2_), 2.91 (2H, broad signal, H_3_), 2.78 (3H, broad signal, H_1_), 2.31 (2H, broad signal, H_3_). This was further functionalized for quaternization using methyl iodide and K_2_CO_3_ in DMSO; that is the copolymer *m*-*p*-Pip-20 (2.75 g, 8.44 mmol) and K_2_CO_3_ (0.58 g, 4.23 mmol) were taken in DMSO in a 25 mL round bottom flask. Methyl iodide (2.63 mL, 42.21 mmol) was then added to this heterogeneous solution and allowed to stir at 80 °C for 48 h. It was observed that the color of the reaction mixture changed from light yellow to dark brown. The reaction mixture was precipitated in a 4:1 ether-isopropyl alcohol mixture several times before filtration. The solid precipitate obtained was filtered and dried in the oven at 80 °C overnight to give poly(*meta*/*para*-terphenylene-methyl piperidinium), *m*-*p*-MP-20, as a brown powder (2.61 g, 95%); *δ*_H_ (400 MHz, DMSO-*d*_6_) 7.80–7.43 (12H, m, H_4,5,6,7,8_), 3.42 (2H, broad signal, H_2_), 3.11 (5H, broad signal, H_2,1_), 2.81 (5H, broad signal, H_3,1_) and 2.46 (2H, broad signal, H_3_).

The same procedure was carried out to synthesize *m*-*p*-MP-50 and *m*-*p*-MP-60 by using 0.5:0.5 and 0.6:0.4 molar ratios of *meta*/*para*-terphenyl comonomers respectively.

### 2.2. Membrane Fabrication

All the membranes based on poly(*meta*/*para*-terphenylene-methyl piperidinium), *m-p*-MP-ys, were prepared by a solution-casting method using the corresponding polymer solution in DMSO as a solvent. A 3 wt.% homogeneous polymer solution was prepared in a glass vial, filtered through a cotton plug under reduced pressure and then poured on a flat glass plate. The membrane film was cast in an oven and dried under vacuum at 80 °C for 24 h to evaporate the solvent completely. The resulting membranes were further treated for hydroxide exchange by immersing them in distilled water for about 4 h to remove the residual solvent from the film. The membranes were then soaked in 1 M KOH solution for 36 h in a closed vial under nitrogen environment at room temperature to exchange the iodide anions for hydroxide ions. Finally, the membranes were washed thoroughly with deionized water prior to any measurements.

### 2.3. Characterization and Miscellaneous Measurements

All the detailed characterizations and various types of measurement techniques were conducted following the previous reports [8,21] and are included in the supporting information.

## 3. Results and Discussions

### 3.1. Polymer Synthesis and Characterization

**Scheme 1:**
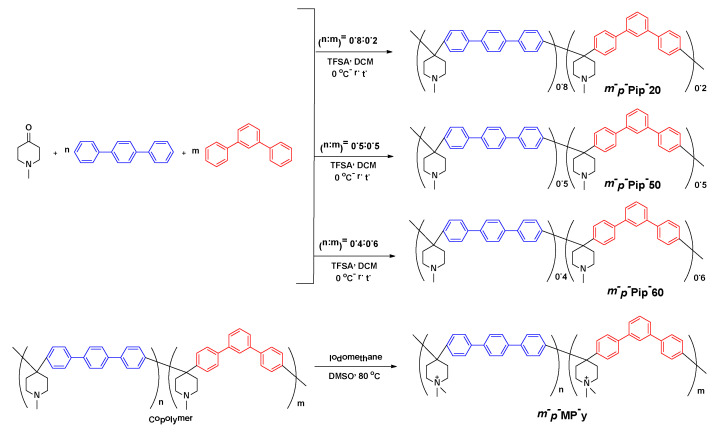
Synthetic route to the poly(*meta*/*para*-terphenylene-methyl piperidinium)s, *m*-*p*-MP-ys.

In the present work, we synthesized poly(*meta*/*para*-terphenylene-methyl piperidinium) copolymers with various contents of *m*-terphenyl units, *m-p*-MP-ys, using Friedel–Craft polymerization between the corresponding *meta/para*-terphenyl monomers and *N*-methyl-4-piperidone as shown in Scheme 1. First, the electron-rich aromatic terphenyl monomers rapidly reacted with ketone in the presence of the TFSA catalyst [16]. The poly(*meta*/*para*-terphenylene-piperidine) copolymers with molar ratios between *meta*-terphenyl and *para*-phenyl comonomer were set to 20:80, 50:50, and 60:40 and were designated as *m-p*-Pip-20, *m-p*-Pip-50, and *m-p*-Pip-60, respectively, where the number (y) in each designation (*m-p*-Pip-y) represents the contents of the *meta*-terphenyl unit in the copolymer.

The structure of all three copolymers (*m-p*-Pip-y)s was confirmed by spectroscopic analysis using ^1^H NMR in DMSO-*d*_6_/Trifluoro acetic acid (TFA) mixture (Figure S1 in Supporting Information), and it was observed that the characteristic peaks of the aromatic protons appeared between 7.91 and 7.36 ppm. The protonation of piperidine by TFA caused a separation and splitting of the piperidine protons; the methylene protons (H_2_ and H_3)_ appeared around 3.52 ppm, 3.22 ppm, 2.91 ppm, and 2.30 ppm. In addition, the peak at 2.78 ppm corresponded to the methyl protons on the nitrogen atom. All these results indicate a successful synthesis of poly(*meta*/*para*-terphenylene-piperidine)s. The compositional analysis of *meta*-phenyl units in the copolymers was, however, not possible, since all the aromatic peaks corresponding to the *meta*- and *para*-terphenyls were overlapped at the range of 7.91 to 7.36 ppm (Figure S1). The feed ratio was, therefore, used for the composition between the *meta*-phenyl and *para*-phenyl unit in the poly(*meta*/*para*-terphenylene-piperidine)s.

The poly(*meta*/*para*-terphenylene-piperidine) copolymers were further functionalized for quaternization using methyl iodide and K_2_CO_3_ to the desired poly(*meta*/*para*-terphenylene-methyl piperidinium)s with three different *meta*-phenyl units. The functionalized polymers were soluble in organic solvents such as *N,N*-dimethylformamide (DMF) and *N*-methyl-2-pyrrolidone (NMP).

Finally, the structure of the poly(*meta*/*para*-terphenylene-methyl piperidinium) after the methylation process was further confirmed by spectroscopic analyses using ^1^H NMR, and it was found that a new characteristic peak appeared around at 3.11 ppm, due to methyl protons from the quaternized piperidinium group, while all other peaks remained almost constant, indicating that the piperidine group was successfully quaternized to methyl piperidinium as a conducting head group (Figure S2).

### 3.2. Membrane Preparation

**Figure 2:**
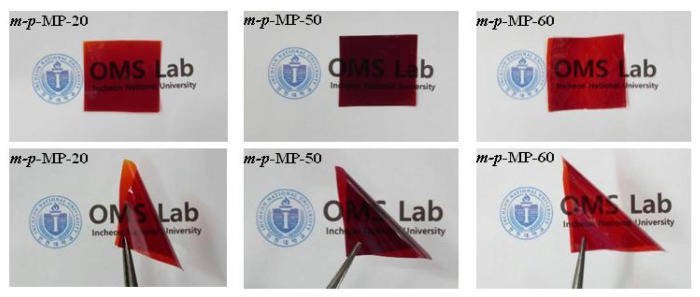
Photographs of the *m-p*-MP-20, *m-p*-MP-50, and *m-p*-MP-60 copolymer membranes.

Membranes based on poly(*meta/para*-terphenylene-methyl piperidinium) copolymers, *m-p*-MP-ys, in their hydroxide (OH^−^) forms with three different *meta*-phenyl units were prepared by the solvent casting method using 3 wt.% solutions of each polymer in DMSO. The prepared brown membranes in their iodide forms were of a thickness controlled to 30 to 40 µm. The immersion of the membranes in a KOH solution finally afforded the desired poly(*meta/para*-terphenylene-methyl piperidinium) copolymer membranes with hydroxide anions. The membranes that formed appeared uniform and flexible (Figure 2).

### 3.3. Morphological Analyses

**Figure 3:**
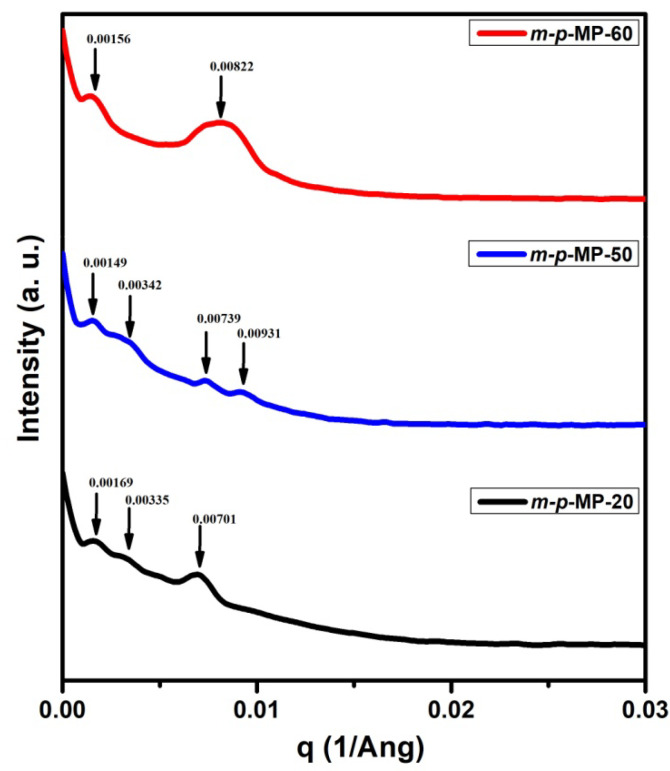
Small angle X-ray scattering (SAXS) profiles of the *m-p*-MP-20, *m-p*-MP-50, and *m-p*-MP-60 copolymer membranes.

The morphologies of the prepared *m-p*-MP-ys were analyzed by small angle X-ray scattering (SAXS) and atomic force microscopy (AFM). At first, clear ionomeric peaks with long range order were observed for all three copolymer membranes (*m-p*-MP-20, *m-p*-MP-50, and *m-p*-MP-60) from the SAXS analysis, indicating a good phase separation between hydrophilic ionic domains and hydrophobic domains (Figure 3). Among three copolymer membranes with different *meta*-phenylene units, the *m-p*-MP-50 membrane with 50% *m*-phenyl showed the lowest *q*-value at 0.00149 Å^−1^, and also the most distinct diffraction patterns, strongly indicating the formation of continuous and well-interconnected ionic channels required for enhanced ionic conductivity. It was suggested that the *meta*-connectivity in terphenyl units permits the polymer backbones to fold back to maximize interactions between the hydrocarbon polymer backbones and enhances the peripheral formation of ion aggregates [39]. Furthermore, the *m-p*-MP-50 membrane also displayed ionomeric peaks at 0.00342, 0.00739, and 0.00931 Å^−1^, indicating the presence of a long-range-ordered morphology [40,41].

In contrast, the *m-p*-MP-60 copolymer membrane with the highest content of the *meta*-phenyl unit showed a strong scattering peak at *q* = 0.00822 Å^−1^ with high intensity, suggesting that a large amount of small ionic clusters were dominant in this membrane and so the continuous ionic channel might not form within it. This may be associated with the aggregation of polymeric chains, due to the largest free volume occupied within the polymer chains [16,42].

**Figure 4:**
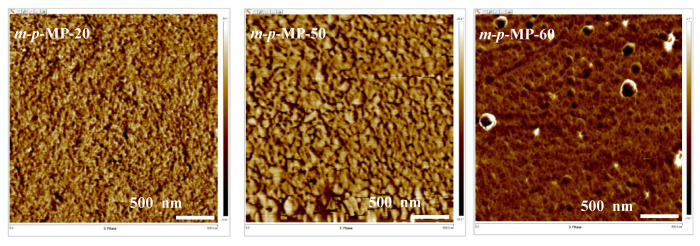
Atomic force microscopy (AFM) phase images of the *m-p*-MP-20, *m-p*-MP-50, and *m-p*-MP-60 copolymer membranes.

The AFM phase images of *m-p*-MP-y copolymer membranes further supported the phase separated morphologies between the dark hydrophilic and the bright hydrophobic domains for all three membranes (Figure 4). It was also found that, as the *meta*-phenyl unit increased from *m-p*-MP-20 to *m-p*-MP-50, the size of the hydrophilic ionic domains increased by peripheral development of hydrophilic ion aggregates, due to the conformational changes brought about by the kink-structured *meta*-phenyl unit [25,43], hence causing the formation of continuous ionic channels. Further increase of the *meta*-phenyl unit to 60 mol% for *m-p*-MP-60, however, caused more aggregation of ionic domains voiding the continuous channel formation, possibly due to the excess free volume brought on by the high content of *meta*-phenyl in the poly(*meta/para*-terphenylene-methyl piperidinium) copolymers.

The AFM height and 3D top view images of the *m-p*-MP-ys further supported the morphological difference in three copolymers (Figures S3 and S4). The *m-p*-MP-20 membrane with only 20% of meta-terphenyl unit showed a rather discrete phase-separation, due to strong interactions between the polymer backbones of the linear *para*-phenyl units which constitute a major part of the copolymers. In contrast, the formation of aggregated ionic clusters was confirmed from the 3D AFM images for the *m*-p-MP-60 having the highest content of the *meta*-phenyl unit: the AFM 3D side view image of this membrane showed large peaks representing the hydrophobic region and troughs representing the hydrophilic region, suggesting the aggregation of the hydrophilic domain (Figure S5).

The results from the AFM analyses were consistent with those of SAXS and indicate that an introduction of *meta*-phenyl unit in the polymer structure of the poly(*meta/para*-terphenylene-methyl piperidinium)s increased the free volume, but there is also an optimum level of this kink-structured unit to achieve well-separated morphology. Overall, the *m-p*-MP-50 with 50% molar ratio of *meta*-phenyl unit showed the best morphology, due to the most balanced spatial configuration between the folded *meta*-terphenyl unit and extended *para*-terphenyl unit, hence both the highest conductivity and the highest dimensional stability are expected for this membrane.

### 3.4. Ion Exchange Capacity, Water Uptake, Swelling Ratio, and Density Measurements of the Membranes

The ion exchange capacity (IEC) of ion-conducting polymers (or ionomers) is defined as the milli-equivalents of cationic groups (conducting head groups) per gram of polymer and has a significant effect on the performance of AEMs. In general, high IEC values of AEMs lead to high conductivity, but at the same time membranes with higher IECs tend to have poor physical properties, including low dimensional stability [44]. The experimental IEC values of the poly(*meta/para*-terphenylene-methyl piperidinium)s were measured by back titration and were found to be in the range of 2.21–2.49 meq·g^−1^. It was also found that the experimental IEC values were almost similar with the theoretical IEC values calculated from the polymer structure in the OH^−^ form, indicating that the poly(*meta*/*para*-terphenylene-piperidine)s were fully quaternized to the desired poly(*meta*/*para*-terphenylene-methyl piperidinium)s.

The water uptake of the AEM is directly related to swelling and ionic conductivity: while high water uptake is generally required for high conductivity, as water is a medium for ion conduction, excessive water uptake inevitably causes swelling (or low dimensional stability) of the membrane [25,45]. Therefore, a moderate water uptake to obtain high conductivity while minimizing swelling is essential for the development of high performance AEMs. The water uptake and swelling ratio of the poly(*meta*/*para*-terphenylene-methyl piperidinium)s were measured at temperatures ranging from 20 °C to 80 °C (Table 1**)**. It was found that the *m-p*-MP-20 showed the least water uptake, due to its linear structural conformation, and the water uptake increased as the *meta*-phenyl unit increased from *m-p*-MP-20 to *m-p*-MP-60, due to the increased free volume created by the increased content of the kink-structured *meta*-terphenyl unit in the copolymers.

As expected, the swelling ratio in the length direction (Δ*l*) also rose with the increase of the *meta*-phenyl unit. It was, however, found that the *m-p*-MP-50 with 50% molar ratio of the *meta*-phenyl unit showed the lowest swelling ratio (or highest dimensional stability) in the thickness direction (Δ*t*), both at low (20 °C) and high (80 °C) temperatures, among copolymers with three different meta-phenyl units. The lower swelling (or higher dimensional stability) of this membrane means that it is possible to more effectively suppress significant hygrothermal stresses that can occur while membranes are subjected to repeated expansion and contraction due to humidity variation under actual cell operating conditions [46]. This high dimensional stability of *m-p*-MP-50 is ascribed to the best-defined phased separated morphology for this membrane, caused by the balanced spatial configuration between the folded *meta*-terphenyl unit and extended *para*-terphenyl unit, as confirmed by the AFM and SAXS.

The density of the *m-p*-MP-ys was further measured, and it was found that this value decreased with the increase of the *meta*-phenyl unit from *m-p*-MP-20 to *m-p*-MP-60, suggesting that the free volume increased with the incorporation of the kink-structured *meta*-phenyl unit in the poly(*meta*/*para*-terphenylene-methyl piperidinium) copolymers (Table 1).

### 3.5. Ion Conductivity

**Figure 5:**
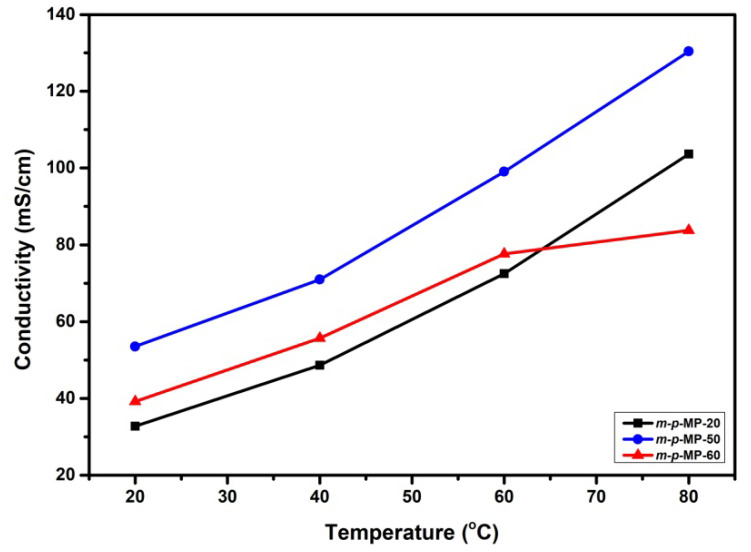
Conductivity profile for the anion exchange membrane (AEM) samples *m-p*-MP-20, *m-p*-MP-50, and *m-p*-MP-60.

Ionic conductivity is the key property for evaluating the AEM performance of fuel cell applications in which high conductivity is generally required to achieve high power density for the AEMFC operation. The conductivity data obtained for the poly(*meta*/*para*-terphenylene-methyl piperidinium)s measured at temperatures ranging from 20 °C to 80 °C are given in Table 2 and Figure 5. Although the *m-p*-MP-y copolymer membranes with all three different *meta*-phenyl units had similar IEC values, the *m-p*-MP-50 copolymer membrane exhibited the highest conductivity of 53.53 mS/cm at 20 °C and 130.39 mS/cm at 80 °C. This is again ascribed to the well-developed interconnected ionic channels originating from the balanced spatial configuration between the linear and rigid *para*-phenyl unit and the folded *meta*-terphenyl unit, as confirmed by the AFM and SAXS. The *m-p*-MP-60 with the highest content of *para*-phenyl showed almost identical conductivity with *m-p*-MP-20 up to 60 °C despite its high water uptake, but this was reversed at 80 °C, and this is ascribed to the dilution of the ionic charge concentration due to the high water content—the so-called “dilution effect” caused by excess water uptake which in turn results from high free volume of the poly(*meta*/*para*-terphenylene-methyl piperidinium) membrane in this composition [47].

In addition, we further compared the hydroxide conductivities of our *m-p*-MP-y membranes with other AEMs prepared from the polyphenylene-type AEMs reported in the recent papers [16,25,26,35,36,48] (Figure S6). It was found that all the *m-p*-MP-y copolymer membranes exhibited similar or higher conductivities than other polymer electrolytes having a similar range of IECs. Among three, the *m-p*-MP-50 showed the highest conductivity, due to the well-separated morphology developed, as mentioned above.

### 3.6. Thermal Stability and Mechanical Properties

**Figure 6:**
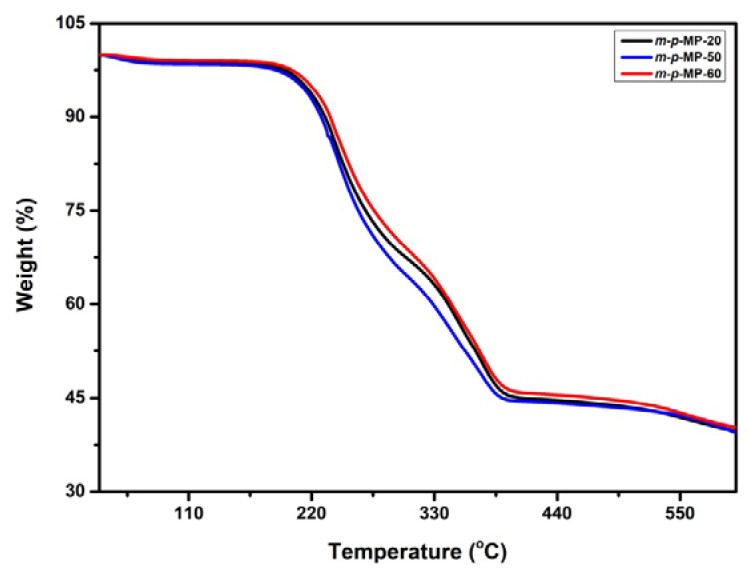
Thermo Gravimetric Analysis (TGA) graphs of the *m-p*-MP-20, *m-p*-MP-50, and *m-p*-MP-60.

The thermal stability of three poly(*meta*/*para*-terphenylene-methyl piperidinium)s were analyzed by thermo gravimetric analysis (TGA) up to 600 °C (Figure 6). The first weight loss observed below 200 °C was ascribed to the evaporation of the residual solvents and water in the membranes. This was followed by two-stage mass losses, where the first degradation between 200 °C and 300 °C was due to the decomposition of piperidinium groups and the second degradation between 300 °C and 450 °C was ascribed to the polymer backbone degradation. Overall, all three membranes showed high thermal stability up to 200 °C, good enough for fuel cell operation. In addition, the differential scanning calorimetry (DSC) analyses of the *m-p*-MP-y copolymer membranes were further carried out to confirm the effect of the backbone structure on the polymer chain mobility of the membranes (Figure S7). As expected, the higher molar ratio of the rigid *p*-phenyl segment brought a higher glass transition temperature (*T_g_*). It can be seen that from *m-p*-MP-20 to *m-p*-MP-60, the *T_g_* decreased from 110.6 °C to 106.0 °C, which confirmed a higher free volume generated in the *m-p*-MP-ys copolymers [42].

**Figure 7:**
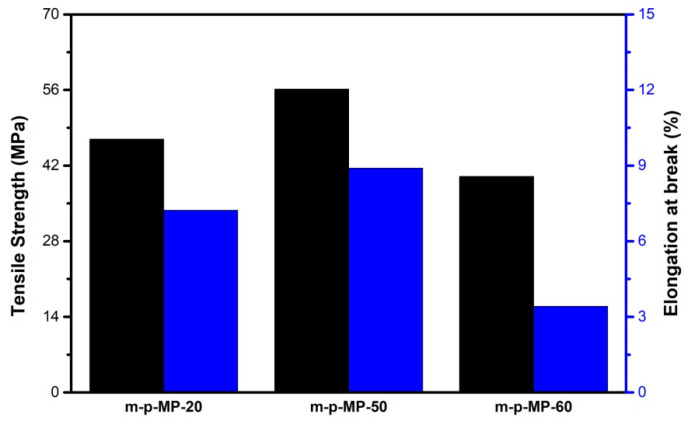
The mechanical properties of the *m-p*-MP-20, *m-p*-MP-50, and *m-p*-MP-60 membranes.

Another important factor for assessing the properties of the developed AEMs is their mechanical property, which was analyzed by monitoring the stress-strain curves of the membranes (Figure 7). The tensile stress for all three membranes was observed in the range of 40.00–56.16 MPa and the elongation at break was from 3.41% to 8.90%. The *m-p*-MP-50 membrane showed the highest tensile strength and elongation at break of 56.16 MPa and 8.90%, respectively. The controlled polymer design, and hence the well-developed morphology, are obviously related with this excellent mechanical property of this membrane. The *m-p*-MP-60 with the highest content of the kink-structured *meta*-phenyl unit showed the lowest tensile strength and elongation at break, due to especially high water uptake by this membrane. These results strongly suggest that too much free volume adversely affects the mechanical property of the AEMs, and there is an optimum level for the conformational structure.

### 3.7. Single Cell Performance

**Figure 8:**
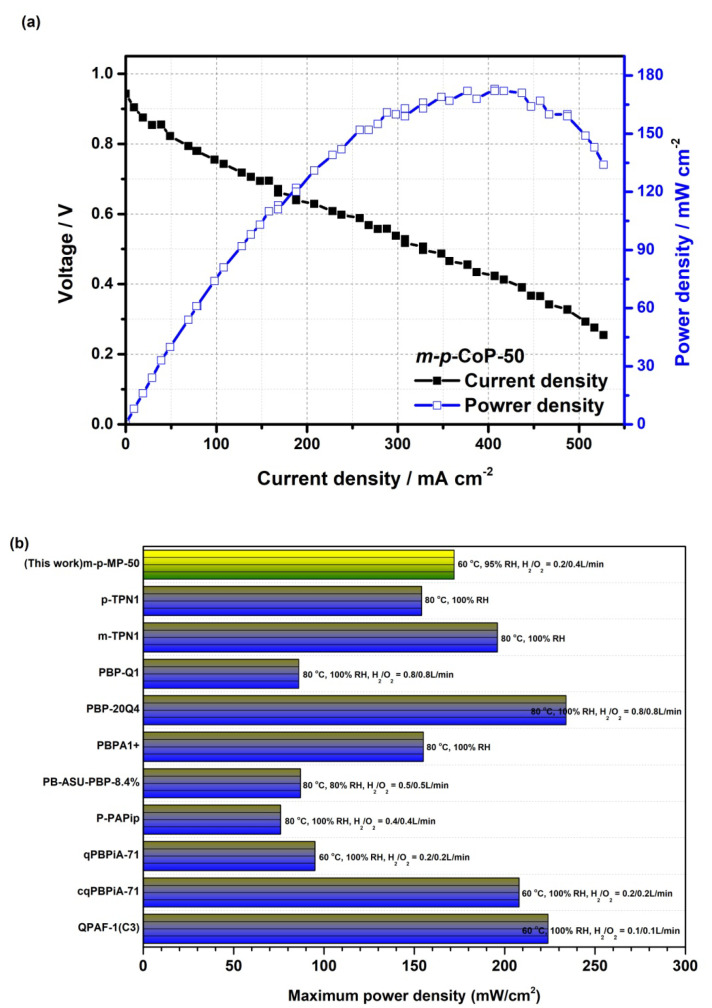
(**a**) H_2_/O_2_ single cell performance data of the *m-p*-MP-50 membrane in 95% RH at 60 °C and (**b**) maximum power density values of recently reported rigid polymer-based AEMs, compared with the *m-p*-MP-50 membrane.

The *m-p*-MP-50 membrane with 50 mol% of meta-phenyl unit was found to be the best among three copolymer membranes with different *meta*-phenylene units in terms of chemophysical and electrochemical properties. With these results in hand, this membrane was further investigated for the single-cell performance for AEMFC application. Figure 8a represents the polarization and power density curves of the corresponding membrane with a thickness of 27 μm performed at 60 °C and 95% RH conditions. The cell test was carried out under H_2_/O_2_ conditions and the copolymer membrane manifested a good fuel barrier ability by attaining an open circuit voltage of 0.943 V [49]. The peak power density (P_max_) achieved for the *m-p*-MP-50 membrane was 172 mW/cm^2^ at a current density of 407 mA/cm^2^. In addition, a current density of 238 mA/cm^2^ at 0.6 V was obtained for this membrane.

Compared to the AEMs based on rigid polymers with aryl ether free backbone, as reported in recent papers [26,27,36,37,39,48,50], the AEM based on *m-p*-MP-50 exhibited moderate to good cell performance (Figure 8b). This cell performance was attributable to the well-separated morphology that resulted from controlling the conformational structure by balancing between the linear *para*-terphenyl and kink-structured *meta*-terphenyl units.

### 3.8. Alkaline Stability

**Figure 9:**
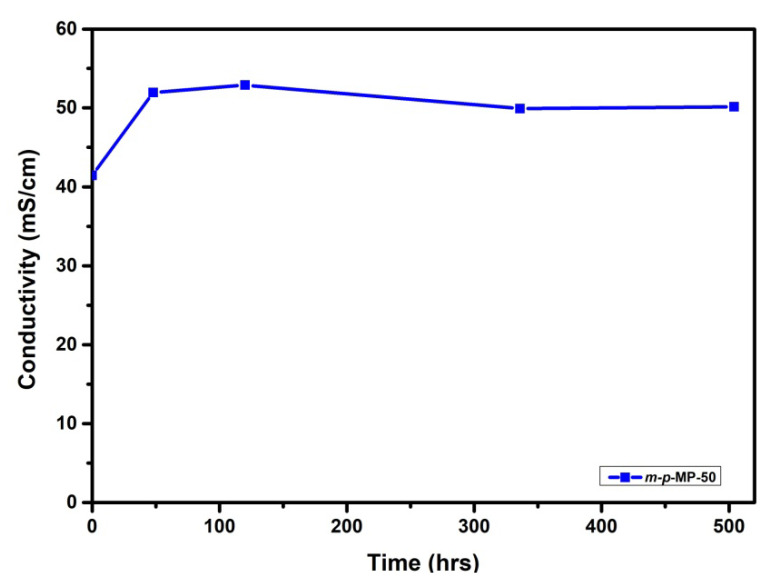
The alkaline resistance of the AEM sample *m-p*-MP-50 in 1 M KOH at 80 °C.

Aside from the physical stabilities such as mechanical and thermal stability of the AEMs, chemical stability in an alkaline condition (or alkaline stability) where AEMFCs operate is also very important to determine the duration of fuel cells. The chemical stability of the *m-p*-MP-50 membrane in an alkaline condition was, therefore, examined by measuring its hydroxide conductivity in a 1M KOH solution at 80 °C for 500 h at a certain time interval in terms of its decrease over time (Figure 9). After a certain period, this membrane was taken out of the KOH solution and carefully washed with deionized water. The hydroxide ion conductivity was then measured at 20 °C. The increment of hydroxide conductivity was observed up to 48 h, and it was attributed to the complete conversion of the conducting head group, that is piperidinium, from the halide form to the hydroxide anion form [8,26]. After this point, this membrane showed conductivity loss less than 4% up to 500 h, indicating long-term chemical stability. The membrane also maintained its original flexibility even after the alkaline test. The combination of non-aryl ether (C–O) linkages and balanced conformational structure between linear *para*-phenyl and kink-structured *meta*-phenyl units in the polymer backbone, together with the piperidium conducting head groups, are the reasons for the excellent chemical stability of the *m-p*-MP-50 membrane.

## 4. Conclusions

In the present study, anion exchange membranes (AEMs) based on poly(*meta/para*-terphenylene-methyl piperidinium) copolymers with different *meta*-terphenyl units were developed. The various characteristics of these AEMs were observed and examined with respect to the composition of the *meta*-terphenyl unit in the copolymers. The meta-connectivity in terphenyl units is expected to allow the polymer chains to fold back to maximize interactions between the hydrocarbon backbones, promoting the peripheral formation of ion aggregates, due to the free volume generated by the kink structure.

The *m-p*-MP-50, having 50 mol% of the *meta*-terphenyl unit, exhibited the highest conductivity while experiencing the least swelling together with the highest mechanical stability among three *m-p*-MP-ys with different compositions. This is due to the well-defined morphology resulting from the controlled free volume, which in turn originates from the balanced conformational structure between the linear *para*-terphenyl and kink-structured *meta*-terphenyl units. The *m-p*-MP-50, with an IEC of 2.49 meq/g, presented a high conductivity of 130.39 mS/cm at 80 °C. Moreover, good mechanical properties and high thermal and alkaline stability (it maintained 96.4% of the initial conductivity even after immersion in 1 M KOH at 80 °C for 500 h) were obtained for this membrane. The *m-p*-MP-50 also showed a peak power density of 172 mW/cm^2^ at a current density of 407 mA/cm^2^ at 60 °C, and these results are either comparable to or better than those for other AEMs based on the rigid polymer backbones reported.

The major findings of the present study show that with the introduction of a kink-structured rigid molecule capable of generating free volume can not only improve the performance of the fundamental properties of AEMs but also their cell performance, which is essential for AEMs to be used in working fuel cells.

## Figures and Tables

**Table 1:** Ion exchange capacity (IEC), water uptake, swelling ratio, and density of the *m-p*-MP-y membranes.

Membrane Code	IEC (meq/g)		Water Uptake (%)		Swelling Ratio (%)				Density (Dry)
	Calc. ^a^	Exp. ^b^	20 °C	80 °C	20 °C (Δl)	80 °C (Δl)	20 °C (Δt)	80 °C (Δt)	
*m-p*-MP-20	2.80	2.21 ± 0.06	45.6 ± 0.4	96.4 ± 0.5	19.0 ± 0.3	28.6 ± 0.6	17.6 ± 0.5	19.0 ± 0.6	1.74
*m-p*-MP-50	2.80	2.49 ± 0.01	49.3 ± 0.7	107.5 ± 1.8	19.0 ± 0.2	33.3 ± 0.3	11.1 ± 0.7	12.5 ± 0.4	1.66
*m-p*-MP-60	2.80	2.30 ± 0.03	59.4 ± 0.8	201.7 ± 2.9	20.0 ± 0.5	45.0 ± 0.4	19.4 ± 0.6	23.7 ± 0.7	1.64

^a^ Theoretical values calculated from the polymer structure in the OH^−^ form. ^b^ Experimental values.

**Table 2:** Conductivity values of *m-p*-MP-y copolymer membranes.

Membrane Code	OH^−^ Conductivity (mS/cm)			
	20 °C	40 °C	60 °C	80 °C
*m-p*-MP-20	32.76 ± 0.12	48.60 ± 0.01	72.50 ± 0.01	103.67 ± 0.71
*m-p*-MP-50	53.53 ± 0.15	70.96 ± 0.37	99.07 ± 0.21	130.39 ± 0.59
*m-p*-MP-60	39.18 ± 0.12	55.67 ± 0.11	77.62 ± 0.30	83.79 ± 1.27

## Supplementary Materials

The following are available online at https://www.mdpi.com/2077-0375/10/11/329/s1, Figure S1: 1H NMR spectra of the *m-p*-Pip-20 (a), *m-p*-Pip-50 (b), and *m-p*-Pip-60 (c) copolymers, Figure S2: 1H NMR spectra of the *m-p*-MP-20 (a), *m-p*-MP-50 (b), and *m-p*-MP-60 (c) copolymers, Figure S3: AFM height images of the *m-p*-MP-20, *m-p*-MP-50, and *m-p*-MP-60 copolymer membranes, Figure S4: AFM 3D images (top view) of the *m-p*-MP-20, *m-p*-MP-50, and *m-p*-MP-60 copolymer membranes, Figure S5: AFM 3D image (side view) of the *m-p*-MP-60 copolymer membrane, Figure S6: Comparison of the conductivity of the *m-p*-MP-y membranes with other polyphenylene-type AEMs at room temperature as a function of IEC, Figure S7: DSC plots of the *m-p*-MP-20, *m-p*-MP-50, and *m-p*-MP-60 membranes.
