# Supplementary material for: Poly(meta/para-Terphenylene-Methyl Piperidinium)-Based Anion Exchange Membranes: The Effect of Backbone Structure in AEMFC Application
Source: Membranes (Basel). 2020 Nov 5;10(11):329. doi: 10.3390/membranes10110329 (PMC7694387; doi:10.3390/membranes10110329)
Supplement: Supplementary file 1 [file membranes-10-00329-s001.pdf]

membranes

Supplementary Materials

Poly(*meta*/*para*-terphenylene-methyl piperidinium)-Based Anion Exchange Membranes: the Effect of Backbone Structure in AEMFC Application

T.S. Mayadevi [1,2,†], Seounghwa Sung [1,2,†], Listo Varghese,[1,2] and Tae-Hyun Kim[1,2,*]

[1] Organic Material Synthesis Laboratory, Department of Chemistry, Incheon National University, 119 Academy-ro, Yeonsu-gu, Incheon, 22012, Republic of Korea; mayadevits24@gmail.com (T.S.M.); tmdghk712@naver.com (S.S.); listovarghese@gmail.com (L.V.)
[2] Research Institute of Basic Sciences, Incheon National University, 119 Academy-ro, Yeonsu-gu, Incheon, 22012, Republic of Korea.
* Correspondence: tkim@inu.ac.kr ; Tel. +82-32-8358232.
† These authors contributed equally to this work.

Section S1

The molecular structure of the copolymers *m-p*-Pip-y and *m-p*-MP-y was confirmed via ^{1}H NMR spectra using an Agilent 400-MR (400 MHz) instrument using tetramethylsilane (TMS) and dimethyl sulfoxide-d_6/TFA as the internal standard and deuterated solvent respectively. The chemical shifts were listed in parts per million (ppm).

The surface morphology of the copolymer membranes was developed by an Atomic Force Microscope in a tapping mode by using a Bruker MultiMode instrument. A silicone cantilever having an end radius <10 nm, and a force constant of 40 N/m (NCHR, nanosensors, f=300 kHz) were used to image the AEM samples at ambient temperature. All the membrane samples were equilibrated with 50% RH for at least 24 h prior to the imaging. Each AFM sample was analyzed under the same conditions for maintaining consistency. AFM phase/height/3D images are provided as recorded without further image processing.

The small angle X-ray diffraction patterns (SAXS) of the dry membranes (dried well in room temperature prior to measurement) were obtained using a Rigaku HR-XRD smartlab diffractometer. SAXS plot was recorded at a scanning rate of 0.1°/min in a 2θ range from 0° to 6° with a Cu-Kα X-ray (λ = 1.54Å).

The mechanical properties of all the AEMs were determined by using a benchtop tensile tester (Shimadzu EZ-TEST E2-L instrument) with a crosshead speed of 5 mm/min at 25 °C under RH 50%. The *m-p*-MP-y membranes have a thickness between 30 and 40 μm. Engineering stress was calculated from the initial cross-sectional area of the sample. The AEM samples were cut into a rectangular shape with 40 mm ×10 mm (total area) and 20 mm ×10 mm (test area).

Thermal stability of the casted membranes was analyzed by the thermogravimetric analysis (TGA) measurements on a Shimadzu TGA-2950 instrument. TGA was operated in a temperature range of 30 °C to 600 °C at a heating rate of 10 °C min^{-1} under the nitrogen atmosphere. The glass transition temperature (Tg) profiles of the membranes were recorded by differential scanning calorimetry (DSC) in a dry state membranes with PerkinElmer DSC 4000.

For measuring the membrane density, membrane samples were weighed in air and in high-purity heptane (HPLC grade) at room temperature, using the buoyancy method based on the Archimedes principle. For measuring density of dried membranes, the samples were dried at 40 ℃ for at least 24h. The corresponding membrane density was calculated by the following equation,

$$\rho = \frac{W_{air}}{W_{air} - W_{heptane}} \times \rho_{heptane}$$

where W_{air} is the weight of the sample membrane in air, $W_{heptane}$ is the weight of the sample membrane in heptane and $\rho_{heptane}$ is the density of heptane, respectively.

The ion exchange capacity (IEC) of all the membranes was determined by the common back titration method. Prior to the measurement, the membranes in OH^- form were equilibrated with 0.01 M HCl standard solution (10 cm^3) for 24 h to neutralize the OH^- ions. Then the residual HCl has undergone back titration with 0.01 M NaOH standard solution by using phenolphthalein as an indicator. The membranes were then dried to measure the corresponding dry weights (W_{dry}, g). IEC (in meq g^{-1}) was calculated as the moles of exchangeable hydroxide ions per gram of the dry weight according to the equation given below,

$$\text{IEC (meq g}^{-1}) = (V_{0NaOH}C_{NaOH} - V_{xNaOH}C_{NaOH})/W_{dry}$$

where V_{0NaOH} and V_{xNaOH} are the volumes of the NaOH consumed during the titration (without and with membranes respectively) and C_{NaOH} is the mole concentration of NaOH.

The % water uptake of the membrane sample was measured after soaking the membranes in distilled water for more than 24 h. They were wiped with a filter paper and then weighed immediately for taking the weight of wet membrane (W_{wet}). The weight of dry membrane (W_{dry}) was taken after drying the wet membrane under a vacuum condition for at least 24 h. The water uptake (%) value was then calculated using the following equation,

$$\text{WU (\%)} = [(W_{wet}-W_{dry})/W_{dry}] \times 100$$

Dimensional change (% swelling ratio) of the membranes was evaluated by immersing the round-shaped membranes in deionized water at 20 °C and 80 °C, respectively, and the swelling ratios were found from both in-plane and through-plane swelling and were calculated using the following equations,

$$\Delta l = \frac{l_{wet} - l_{dry}}{l_{dry}} \times 100\,, \qquad \Delta t = \frac{t_{wet} - t_{dry}}{t_{dry}} \times 100$$

where l_{dry} and t_{dry} are the diameter and thickness of the dried membranes, respectively, and l_{wet} and t_{wet} refer to those of the membranes after soaking in water for 24 h. The membranes were dried by placing those under vacuum at 40 °C for 4 h prior to the measurement.

Hydroxide conductivity (σ) of each AEM sample (size: 1 cm ×4 cm) was calculated using the equation, $\sigma = l/RA$; where, l refers to the distance between reference electrodes and A is the cross-sectional area of a membrane coupon. The ohmic resistance (R) was recorded by four-point probe alternating current (AC) impedance spectroscopy. The electrode systems were connected with an impedance/gain-phase analyzer (SI-1260) and an electrochemical interface (SI-1287) over the frequency range from 100 mHz to 2 MHz. The conductivity measurements were performed from 20 ^{o}C to 80 ^{o}C. The membrane sample in a cell was immersed in deionized water in a chamber to keep the relative humidity at 100%. The conductivity value was then obtained by the average of at least 3 trials with same time intervals.

The durability of the membranes was evaluated by monitoring the changes in hydroxide ion conductivity by soaking the *m-p*-MP-50 copolymer membrane (OH^- form) into 1 M KOH solution at 80 °C for 500 h. In every interval of time, each sample was taken out and washed with deionized water for several times to wash out the residual KOH inside the membrane, and then soaked in deionized water for at least 4 h at room temperature until neutralization. Then conductivity of each membrane was measured at room temperature in pure deionized water, using the same method mentioned above.

Single cell test was carried out using the best performed *m-p*-MP-50 membrane. For the measurement, the catalyst inks were prepared by mixing Pt/C catalysts (Tanaka, 46.2 wt%), deionized water, n-propanol and 10 wt% ionomer solution (Fumatech FAA ionomer). As prepared inks were coated the membrane with an air spray gun to prepare the Catalyst-Coated Membranes (CCMs). The Membrane Electrode Assemblies (MEAs) were sandwiched between gas diffusion layers (Sigracet 39BC) and gaskets (Teflon®) with an effective electrode area of 5 cm^2. Fuel cell tests were operated at 60 °C and 95% RH and H_2/O_2 were fed into the cell at flow rates 200 cc min^{-1} and 400 cc min^{-1}, respectively. The MEA activation time was 3 h to ensure the steady state.

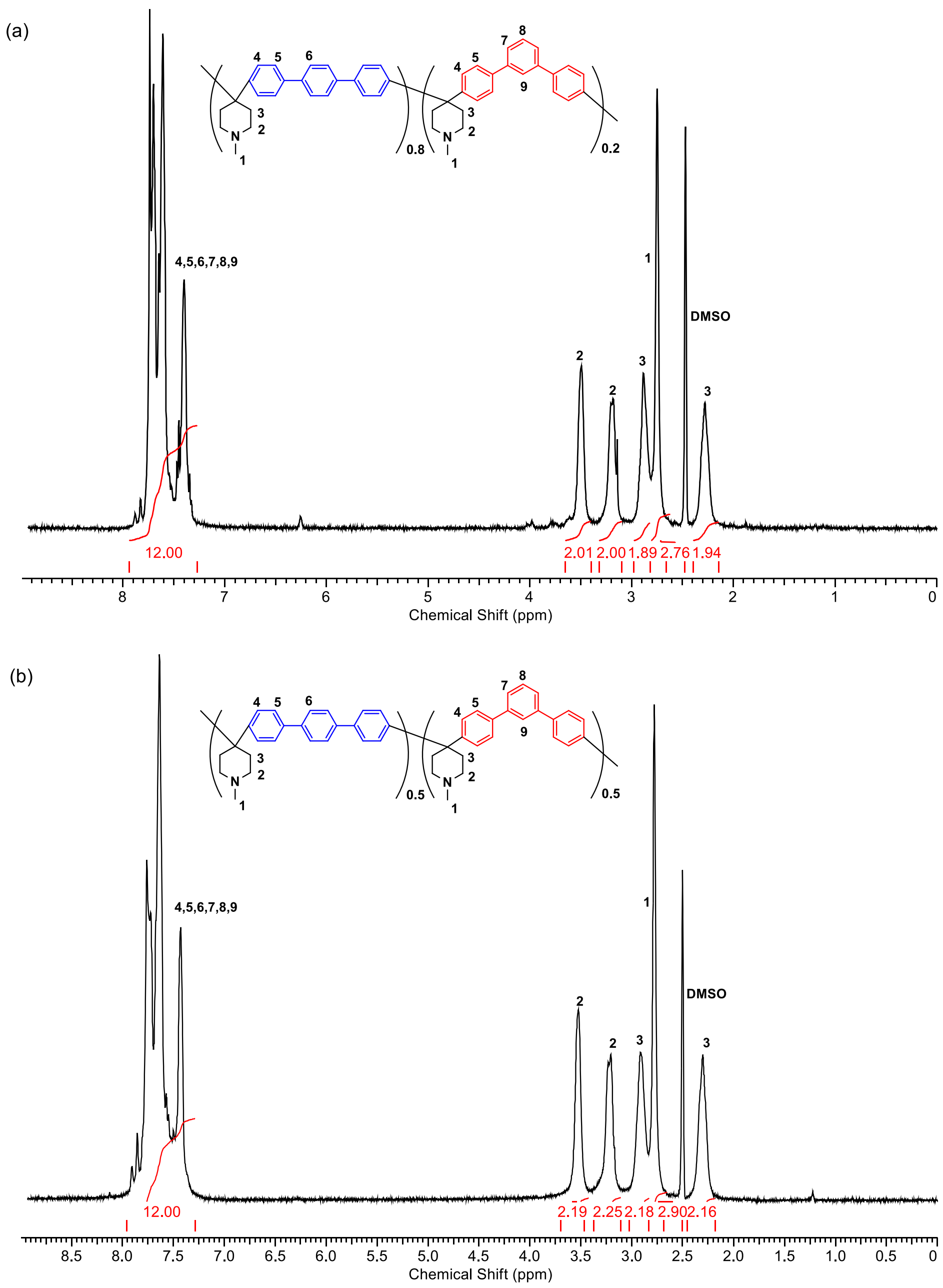

(a)
4,5,6,7,8,9
1
DMSO
2
2
3
3
12.00
2.01
2.00
1.89
2.76
1.94
0.8
0.2
Chemical Shift (ppm)
(b)
4,5,6,7,8,9
1
DMSO
2
2
3
3
12.00
2.19
2.25
2.18
2.90
2.16
0.5
0.5
Chemical Shift (ppm)

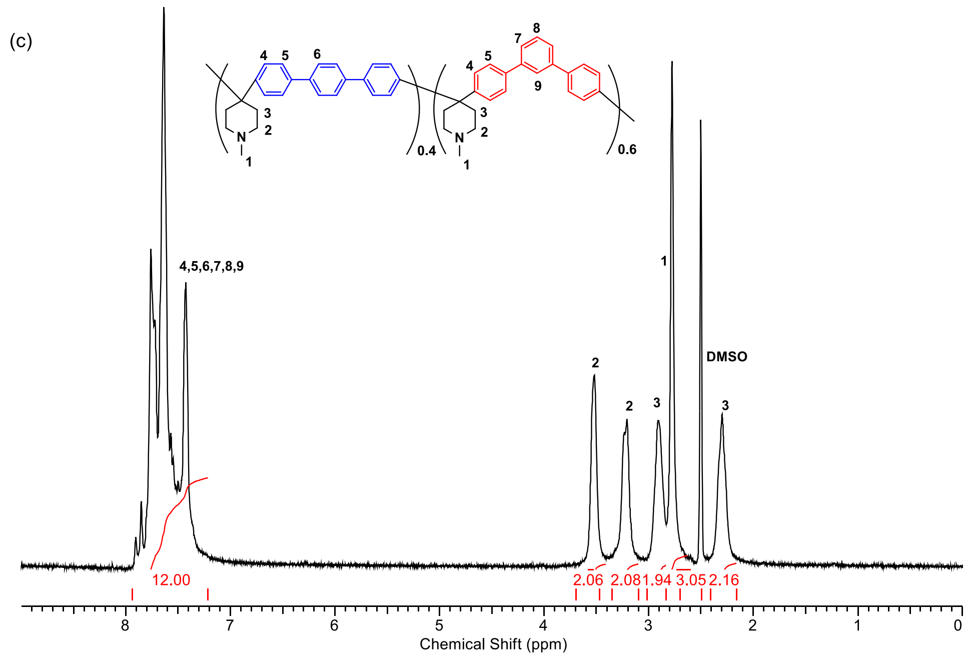

Figure S1. ^{1}H NMR spectra of the *m*-*p*-Pip-20 (a), *m*-*p*-Pip-50 (b), and *m*-*p*-Pip-60 (c) copolymers.

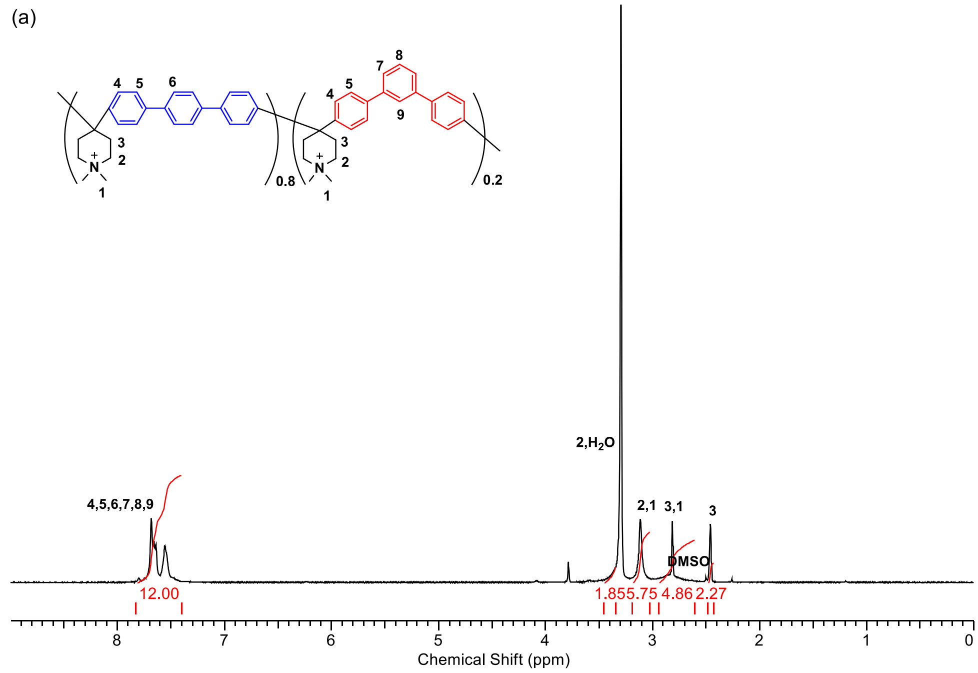

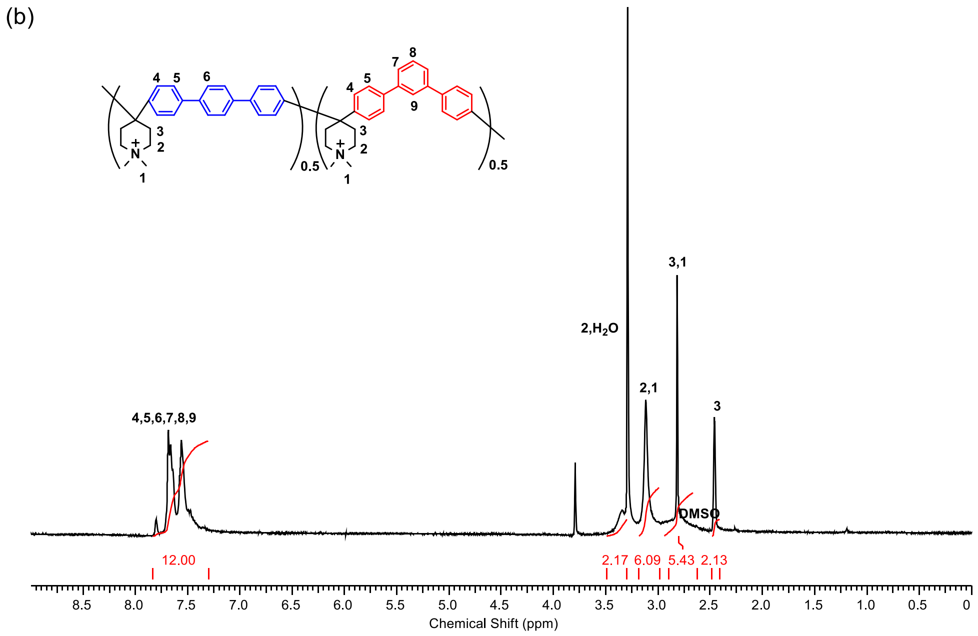

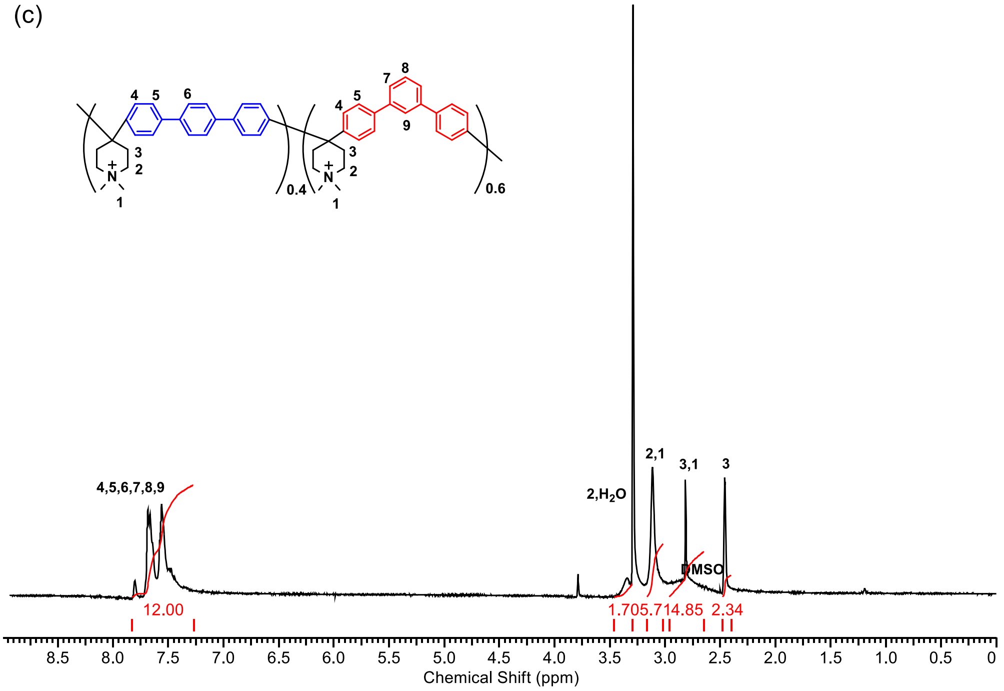

Figure S2. ^{1}H NMR spectra of the *m-p*-MP-20 (a), *m-p*-MP-50 (b), and *m-p*-MP-60 (c) copolymers.

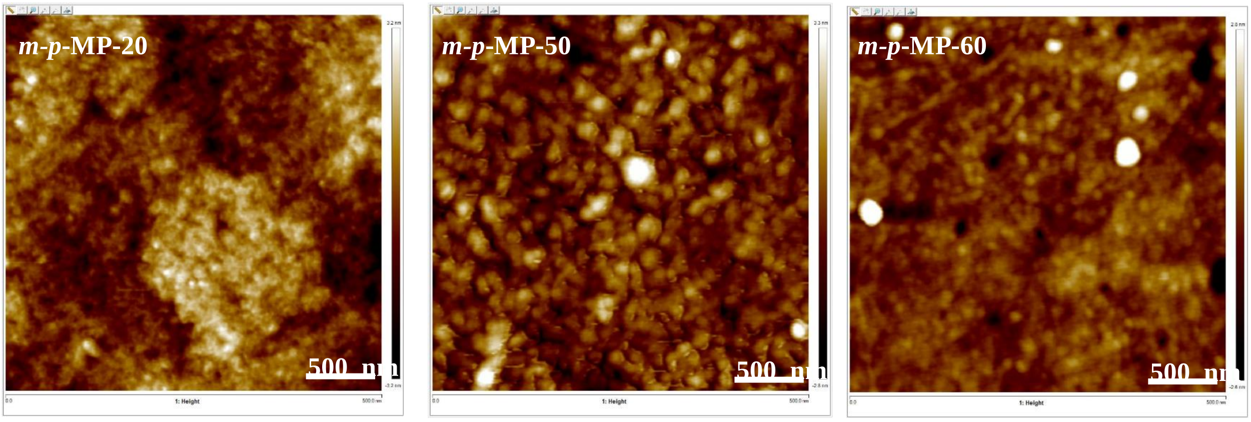

Figure S3. AFM height images of the *m*-*p*-MP-20, *m*-*p*-MP-50, and *m*-*p*-MP-60 copolymer membranes.

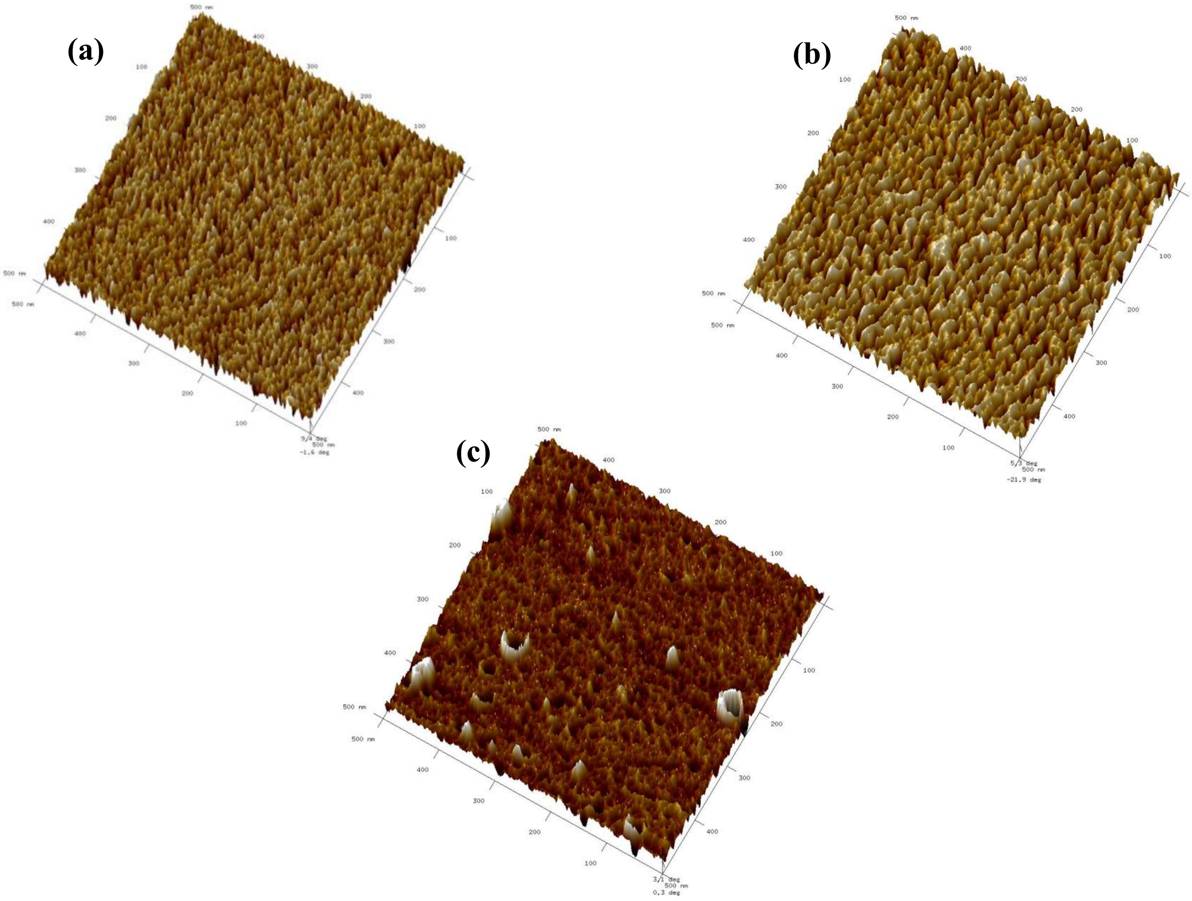

Figure S4. AFM 3D images (top view) of the *m*-*p*-MP-20, *m*-*p*-MP-50, and *m*-*p*-MP-60 copolymer membranes.

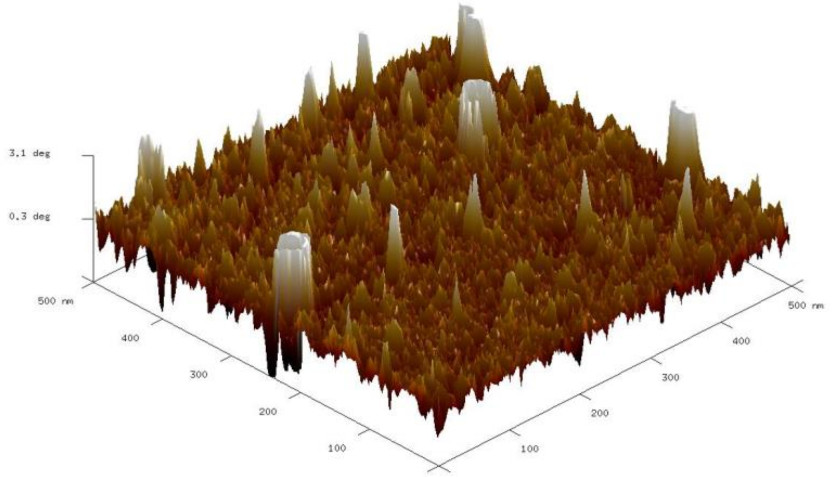

Figure S5. AFM 3D image (side view) of the *m*-*p*-MP-60 copolymer membrane..

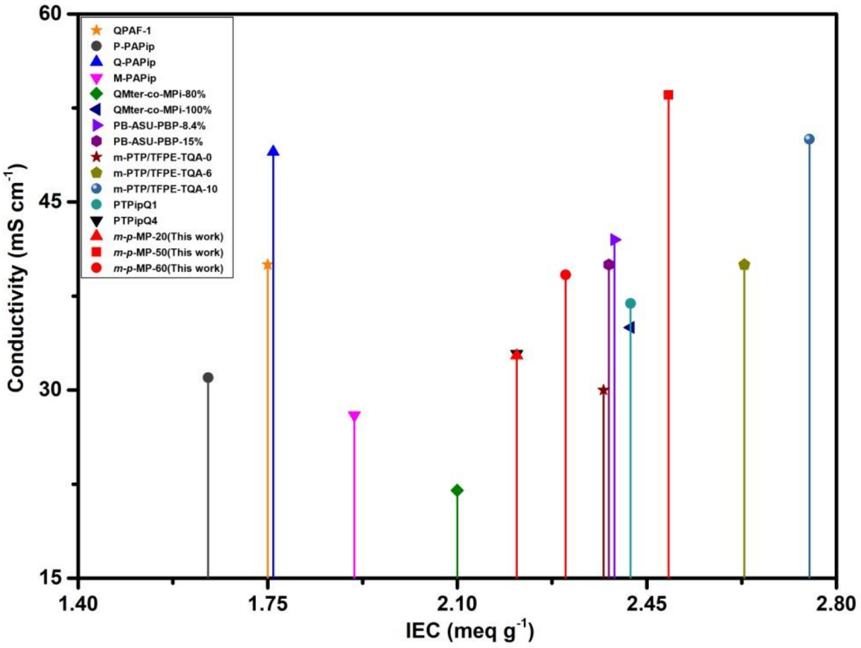

Figure S6. Comparison of the conductivity of the *m*-*p*-MP-y membranes with other polyphenylene-type AEMs at room temperature as a function of IEC.

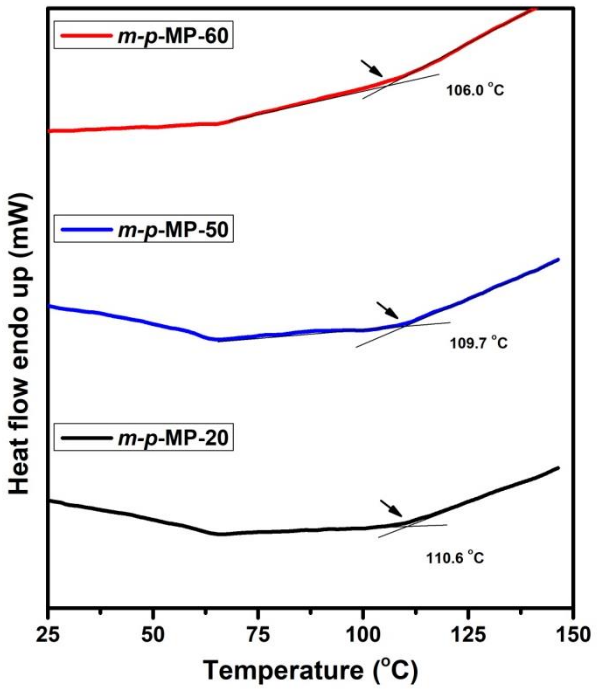

Figure S7. DSC plots of the *m*-*p*-MP-20, *m*-*p*-MP-50, and *m*-*p*-MP-60 membranes.
